# Effect of Transforming Growth Factor-β upon *Taenia solium* and *Taenia crassiceps* Cysticerci

**DOI:** 10.1038/s41598-017-12202-z

**Published:** 2017-09-27

**Authors:** Laura Adalid-Peralta, Gabriela Rosas, Asiel Arce-Sillas, Raúl J. Bobes, Graciela Cárdenas, Marisela Hernández, Celeste Trejo, Gabriela Meneses, Beatriz Hernández, Karel Estrada, Agnes Fleury, Juan P. Laclette, Carlos Larralde, Edda Sciutto, Gladis Fragoso

**Affiliations:** 1 Instituto Nacional de Neurología y Neurocirugía, Mexico, D.F. 14269 Mexico; 2 Unidad Periférica para el Estudio de Neuroinflamación en Patologías Neurológicas del Instituto de Investigaciones Biomédicas en el Instituto Nacional de Neurología y Neurocirugía, Mexico, D.F. 14269 Mexico; 3 Facultad de Medicina, Universidad Autónoma del Estado de Morelos, Cuernavaca, Morelos 62350 Mexico; 4 Instituto de Investigaciones Biomédicas, Universidad Nacional Autónoma de México, Mexico, D.F. 04510 Mexico; 5 Facultad de Medicina, Universidad Nacional Autónoma de México, Mexico, D.F. 04510 Mexico; 6 Instituto de Biotecnología, Universidad Nacional Autónoma de México, 62250 Cuernavaca, Morelos Mexico

## Abstract

Taeniids exhibit a great adaptive plasticity, which facilitates their establishment, growth, and reproduction in a hostile inflammatory microenvironment. Transforming Growth Factor-β (TGFβ), a highly pleiotropic cytokine, plays a critical role in vertebrate morphogenesis, cell differentiation, reproduction, and immune suppression. TGFβ is secreted by host cells in sites lodging parasites. The role of TGFβ in the outcome of *T. solium* and *T*. *crassiceps* cysticercosis is herein explored. Homologues of the TGFβ family receptors (*TsRI* and *TsRII*) and several members of the TGFβ downstream signal transduction pathway were found in *T*. *solium* genome, and the expression of Type-I and -II TGFβ receptors was confirmed by RT-PCR. Antibodies against TGFβ family receptors recognized cysticercal proteins of the expected molecular weight as determined by Western blot, and different structures in the parasite external tegument. *In vitro*, TGFβ promoted the growth and reproduction of *T*. *crassiceps* cysticerci and the survival of *T*. *solium* cysticerci. High TGFβ levels were found in cerebrospinal fluid from untreated neurocysticercotic patients who eventually failed to respond to the treatment (*P* = 0.03) pointing to the involvement of TGFβ in parasite survival. These results indicate the relevance of TGFβ in the infection outcome by promoting cysticercus growth and treatment resistance.

## Introduction

*Taenia solium* is a parasite whose larval stage (cysticercus) may locate in the human central nervous system, causing neurocysticercosis (NC) a disease prevalent in developing countries. NC may adopt different forms: the clinically mild forms, either asymptomatic or causing few symptoms, and the clinically severe forms, causing a life-threatening, often fatal and frequently disabling form of the disease. Cysticerci may also lodge in the skeletal muscle of the pig (the intermediary host) as a obligated step in the parasite life cycle^1^. *Taenia crassiceps*, a cestode closely related to *T*. *solium*, has allowed us to determine the relevance of parasite-related factors on the infection. Both *T*. *solium* and *T*. *crassiceps* cysticerci can survive in their respective intermediate hosts for years, despite the harmful effect of the inflammatory response they promote^2,3^. Previous studies pointed out the possibility that host immunological and hormonal factors modulate parasite growth and development in various infections^4,5^. In fact, the available evidence increasingly supports that immune-hormonal factors influence several helminth infections through the Transforming Growth Factor-β (TGFβ)^6^ and Epidermal Growth Factor (EGF)^7^; in addition, both molecules are required for androgen and estrogen synthesis^8^ and for insulin to bind peptides through a tyrosine kinase receptor of the insulin receptor family^9^, which also modulates several parasite infections.

TGFβ, a member of a large family of growth factors expressed in both vertebrate and invertebrate cells, is a multifunctional protein, showing a wide variety of effects. TGFβ is secreted as an inactive form, bound to extracellular proteins and then transformed into an active ligand by proteolytic cleavage^10^. A general model for TGFβ signal transduction starts with a complex of transmembranal serine-threonine kinase receptors. Once Type-II kinase receptor binds the TGFβ ligand, it recruits and phosphorylates the Type-I receptor, triggering complex downstream signal transduction pathways^11^.

TGFβ has been found expressed in brain granuloma cells from NC patients^12^, suggesting that this cytokine could be exerting immunomodulatory effects. In addition, it has been observed that some helminths have the potential to produce TGFβ family products (Activin and Bone morphogenetic protein)^6,13–15^ and several of the TGFβ signaling pathways factors have been found in the genome of these parasites^16^. Overall, these findings suggest a relevant role for this growth factor in the host-parasite relationship.

In this study, genes coding for proteins of the TGFβ signaling pathway were searched in *T*. *solium* genome; then, their functional impact on the host-parasite relationship in cysticercosis was studied by measuring their capacity to modulate the growth and survival of *T*. *crassiceps* and *T*. *solium* cysticerci. Additionally, this work explores the possible involvement of TGFβ in the resistance to cysticidal treatment in severe NC human patients.

## Materials and Methods

### Ethics Statement

Human samples: This study fulfilled all research regulations for human beings as required by Mexican laws and international regulations. The Ethical Committee of the Instituto Nacional de Neurología and Neurocirugía (INNN) approved this protocol (Protocol license 133/10). All patients were adult and provided informed consent for sample collection and analysis, and for data publication. All data were anonymized for publication, and no information herein reported could lead to patient identification.

Animal research: All animal protocols followed the guidelines published in the National Institutes of Health Guide for the Care and Use of Laboratory Animals, and were reviewed and approved by the Ethical Committee for the Care and Use of Laboratory Animals (Protocol Number of acceptance: ID 144; Permit Numbers 114 and 115) at the Instituto de Investigaciones Biomédicas, Universidad Nacional Autónoma de México.

All methods were performed in accordance with the guidelines detailed in the National Institutes of Health Guide for the Care and Use of Laboratory Animals, in the Mexican Official Regulation NOM-062-ZOO-2001, and in the International Ethical Guidelines for Biomedical Research Involving Human Subjects, Council for International Organizations of Medical Sciences.

### Mice

BALB/cAnN (AnN) mice were obtained from our animal facility. Original stocks came from Harlan Laboratories (Mexico City, Mexico). The experiments herein reported were performed in young (5 weeks-old) female mice. Mice were bred at our institution’s pathogen-free vivarium, housed in cages (five mice per cage) under controlled temperature and light/darkness cycles, and were allowed food and water *ad libitum*.

### Parasites and Infection

The ORF strain of *T*. *crassiceps* cysticerci was employed, considering its high reproduction rate in BALB/cAnN female mice. Cysticerci used for *in vitro* culture experiments were harvested from the peritoneal cavity of donor BALB/cAnN female mice after 3 months of infection with 20 small (2-mm diameter), non-budding *T*. *crassiceps* cysticerci.

*T*. *solium* cysticerci were obtained from naturally infected pigs from villages in Guerrero, an endemic region for cysticercosis in Mexico. Pigs were euthanized according to ethical veterinary laws in Mexico. Cysticerci were individually harvested from muscle tissue and washed in PBS.

*T*. *solium* and *T*. *crassiceps* cysticerci were cultivated in RPMI 1640 10%-FCS medium (Gibco BRL, Grand Island, NY) for 3 days before use, to remove host molecules. At this time of culture, no anti-cysticercal immunoglobulins were detected by ELISA (data not shown) in cysticercal extracts, an indication that harvested cysticerci were mostly free of host immunological molecules.

### Cysticercal Antigens

*T*. *solium* soluble extract was prepared by centrifuging intact cysticerci at 25,000 × *g* for 60 min at 4 °C, as previously described^17^. Deposited material was discarded, and supernatant, containing a mixture of soluble antigens, was recovered and filter-sterilized.

### TGFβ Family Receptor Gene Expression

*T*. *solium* and *T*. *crassiceps* cysticerci were obtained as described above. RNA was purified from one *T*. *solium* cysticercus or five *T*. *crassiceps* cysticerci with the Rneasy Mini Kit (Qiagen, Hilden, Germany) following the manufacturer’s directions. One-hundred-nanograms of RNA were transcripted to cDNA using the Transcriptor First Strand cDNA Synthesis Kit (Roche, Basel, Switzerland).

PCR reactions were performed using the SsoFast EvaGreen Supermix with low ROX kit (Bio-Rad, San Francisco, CA) according to the manufacturer’s directions. Briefly, 1 µL of the obtained cDNA, 10 µL of the SsoFast Kit and 500 nmol of each reverse and forward primer per reaction (20 µL for each reaction) were used to determine the expression of Type-I and -II TGF-β, and of actin (positive control). The primers used were: Actin (Forward 5′-CGGGTATCCACGAGTCTACTTT-3′ and reverse 5′-TTGATCTTCATGGTGCTTGGC-3′); TGF-βR1 (forward 5′-GGCAACGATGAGAGATGGCT-3′ and reverse 5′-AGGCGATGTGTGTAACGAGG-3′); and TGF-βR2 (forward 5′-GGACTATTTGGCCTTCGGCT-3′ and reverse 5′-AGTCTCTGTGCGCTATGCTC-3′). TGFβ family receptor primers were designed according to the sequences found in *T*. *solium* genome TGFβ Type-I (TsM_001248300) and activin/TGFβ receptor Type 2 A (TsM_000641800). The actin beta-gamma gene was used as a positive control (TsM_000357600). Sample amplification was carried out as follows: template denaturing at 95 °C; annealing at 58 °C for TGF-β receptor 1 and 60 °C for TGF-β receptor 2 and actin; and extension at 72 °C. Each temperature was set for 30 seconds through 40 cycles. Pre-denaturing template and post-extension steps were run for 5 minutes. Amplicons were resolved in 1.5% agarose gels for visualization.

### Immunolocalization of Type-I and -II TGFβ Receptors

To evaluate the ability of antibodies against host-TGFβ receptor to recognize the putative parasite TGFβ receptors, which could point to the potential of TGFβ in promoting parasite growth through specific receptors (as observed in other parasites)^4^, *T*. *crassiceps and T*. *solium* cysticerci slides were searched for TGFβ receptors by cross-immune-reactivity. Cysticerci were fixed and stained following a procedure previously described^18^. Rabbit anti-TGFβRI (H100) and anti-TGFβRII (H-567) polyclonal antibodies (Santa Cruz Biotechnology Inc., Santa Cruz, CA) were employed as primary antibodies. Non-specifically bound host proteins were dissociated from cysticerci using a procedure previously described^18^, followed by incubation in Zamboni solution, pH 7.4 (1.6% w/v paraformaldehyde, 19 mM KH_2_PO_4_, and 100 mM Na_2_HPO_4_•7H_2_O in 240 mL of saturated picric acid and 1,600 mL of H_2_O), for 72 hours. Afterwards, specimens were embedded in paraffin and 6-μm sections were cut. Sections were placed on poly-L-lysine (Sigma, St. Louis, MO)-treated microslides. Peroxidase activity was blocked by treatment with 3% H_2_O_2_ in PBS for 10 min at room temperature. After washing with PBS, sections were blocked with 5% BSA in PBS plus 0.1% Triton X-100 (pH 7.4) for 1 h at 37 °C. Solutions were removed and the slides were incubated overnight at 4 °C with non-immune or specific antisera at a dilution of 1:1000 in 1% BSA in PBS, plus 0.1% Triton X-100 (PBS/A-T). Slides were then treated with primary rabbit anti-TGFβRI or anti-TGFβRII polyclonal antibodies at a 1:200 dilution. After washing three times in PBS/A-T for 5 min each, the slides were covered with biotinylated goat anti-rabbit IgG (Immuno Universal Kit, MP Biomedicals, Santa Ana, CA) for 30 min at 37 °C, rinsed with PBS/A-T, and treated with streptavidin-peroxidase conjugate (Universal Kit, MP Biomedicals, Santa Ana, CA) for 30 min at 37 °C. Peroxidase activity was visualized by incubating the samples with 3′3-diaminobenzidine (DAB-Plus Kit, Zymed, San Francisco, CA). Slides were counterstained with Mayer’s hematoxylin and observed under an optical microscope (Nikon) using the MetaMorph Imaging System, v. 4.5. (Universal Imaging, Downingtown, PA).

### Inhibition of TGFβ Receptor Type-I and -II-Antibody Recognition by TGFβ Binding

*T*. *crassiceps* cysticerci obtained as described above were extensively washed to remove host proteins. Briefly, cysticerci were treated with 500 µL of 50 mM glycine, 0.15 mM NaCl, and 0.1% Triton X-100 for 30 s, followed by the addition of 100 µL of Tris-HCl for another 30 s. Afterwards, cysticerci were washed thrice with PBS and twice with RPMI. Thereafter, 2-mm-diatmeter cysticerci were placed in 96-well, flat-bottomed plates (10 cysticerci per well) and incubated either with PBS or different TGFβ concentrations (0.1, 1, or 10 ng/mL) for 30 min at 37 °C. Then, cysticerci were fixed in buffered formaldehyde and prepared for immunohistochemical staining for TGFβ Type-I and -II receptors, as described above. The intensity of bound antibodies for each receptor was measured using the MetaMorph Imaging system, v.4.5. (Universal Imaging, Downingtown, PA).

### Western Blot

To identify the presence of TGFβ Type-I and -II receptors in *T*. *solium* and *T*. *crassiceps* cysticerci, protein extracts were prepared using the RIPA Lysis and Extraction Buffer (Sigma, St. Louis, MO) according to the manufacturer’s directions, using the complete Mini Protease Cocktail Inhibitor (Roche, Indianapolis, IN). After centrifuging at 10,000 rpm for 20 min at 4 °C to remove debris, protein concentration was determined using the Lowry’s method. Twenty-micrograms of protein were denatured by boiling for 5 min in sample buffer and resolved by reducing SDS-NuPAGE (4–12% (w/v) acrylamide; Invitrogen, Grand Island, NY). Gels were electrophoretically transferred to a nitrocellulose membrane (Bio-Rad, Hercules, CA) using transfer buffer (Tris 0.025 M, pH 8.5; glycine 0.193 M; methanol 20%) in a semi-dry apparatus. Membranes were blocked for 1 h in blocking buffer TBS (Tween 0.1%, BSA 3%). After wash with TBS 0.1%-Tween, primary antibodies anti-TGFβRI or anti-TGFβRII (H-100 or H-1700, Santa Cruz Biotechnology, Santa Cruz, CA) were incubated at a 1:500 dilution in blocking buffer overnight at 4 °C. After washing with TBS 0.1%-Tween, the secondary antibody, goat anti-rabbit IgG HRP (Zymed, Rockford, IL), was incubated at a dilution of 1:5000 in blocking buffer. Bands were detected using TBS blot substrate (50 nm Tris, 250 mM NaCl, pH 7.2) (Zymed, Rockford, IL) after incubation for 10–15 min at room temperature. Protein extracts from 10^6^ PBMCs from healthy subjects were employed as positive control. To determine the specificity of the recognition of TGFβRI or TGFβRII parasite receptors, a control was included in which membranes with cysticercal antigens, were first incubated with a Rabbit IgG anti-Mouse antibodies (Thermo Scientific) followed by goat anti-rabbit IgG as secondary antibody.

### *In Vitro* Effect of TGFβ upon Cysticerci Growth and Survival

Cysticerci were cultured with different TGFβ concentrations to assess the effect of this cytokine upon parasite growth and survival. Experiments were performed by culturing either five non-budding *T*. *crassiceps* cysticerci (2 mm in diameter) or four viable *T*. *solium* cysticerci placed in 1- and 2 mL, respectively, of RPMI 1640 (Gibco BRL, Grand Island, NY) supplemented with penicillin/streptomycin (Gibco BRL, Grand Island, NY), with different concentrations of recombinant human TGFβ1 (eBioscience, San Diego, CA): 0, 0.001, 0.01, or 0.1 ng/mL. As a positive control, parasites were cultured in RPMI 1640 supplemented with 10% fetal bovine serum (FBS) (Gibco BRL, Grand Island, NY). Every other day of culture, 500 μL (for *T*. *crassiceps*) or 1 mL (for *T*. *solium*) of medium were replaced by fresh medium. Cysticerci were microscopically observed at day 0, 6, 13, and 20 for *T*. *crassiceps*, and at day 0, 5, 10, 15, and 20 for *T*. *solium*. Cysticercus viability was assessed by parasite motility, a good indicator of viability, as demonstrated in a previous study which compared temperature-induced motility and vital staining with 0.02% trypan blue^19^.

In *T*. *crassiceps*, follow-up time was set to the moment when the number of buds per cysticercus could be easily determined. The size and viability of both cysticerci species and the number of buds in each *T*. *crassiceps* cysticercus were also microscopically evaluated. Size was measured using the MetaMorph Imaging System v. 4.5. This software triangulates the whole threshold surface (it traces millions of triangles on each object) and then it calculates the area of each triangle; finally, it adds up these areas. A comparison between groups was made, using 0 ng/mL of TGFβ as a control.

### Neurological Patients

A total of 48 patients (76 CSF samples) attending the Instituto Nacional de Neurología y Neurocirugía (INNN) in Mexico City from 2007 to 2010 were included in this study. All patients included showed multiple vesicular cysticerci in the subarachnoid space of the base (SAB) of the brain. Neurocysticercosis (NC) patients were classified according to their response to cysticidal treatment, either as responder (R) or as non-responder (NR). Those patients who showed a reduction in the size or number of parasites greater than 50% were regarded as R, while those showing a reduction in parasite load equal to or less than 50% were regarded as NR. More than 50% of patients (*n* = 27) had received several cycles of cysticidal drugs and corticosteroids. All patients were treated with standard albendazole and corticosteroid doses (albendazole, 30 mg/kg/day for 1 week, and dexamethasone 0.3 mg/kg/day for 1 week, and then prednisone for tapering drug schedule).

### Cytokine Titration by ELISA

CFS samples were stored at −80 °C until use for cytokine quantification. All assays were performed in duplicate, and the sensitivity of the TGFβ ELISA was 9.4 pg/mL.

Sandwich ELISAs were performed in 96-well, flat-bottomed microtiter plates (Nunc-Immuno Plate Maxisorp, Roskilde, Denmark). Microplates were coated for 18 h at 4 °C with the capture antibody (eBioscience, San Diego, CA), washed three times with PBS-Tween 20 (0.05%), blocked for 30 min at room temperature with 2% PBS-BSA, and washed three times. All samples were treated to activate latent TGFβ to its immuno-reactive form. Twenty-microliters of 1 N HCl were added to 100 μL of each sample; samples were incubated for 10 min at room temperature and then neutralized with 20 µL of 1 N NaOH. The plates were then incubated at room temperature for 2 h. After washing, plates were incubated with the detection antibody (eBioscience, San Diego, CA) for 2 h at room temperature. Bound antibodies were detected using streptavidin-phosphatase conjugate (1:3000; Zymed Laboratories, San Francisco, CA) and p-nitrophenyl phosphate (Sigma, St. Louis, MO) as a substrate. Optical density readings were performed at 405 nm, after 30 and 60 min of incubation.

### Statistical Analysis

The mean number of buds per cysticercus was compared using the Student’s unpaired *t-*test, and when values were not normally distributed they were compared using the Mann-Whitney U-Wilcoxon rank non-parametric test. Differences were considered statistically significant at *P* < 0.05.

The viability of *T*. *solium* cysticerci was evaluated using size and motility as indicators. Survival percent was calculated using the Kaplan-Meier estimate. Briefly, survival percent was given by [(Number of cysticerci living at the start of culture – Number of cysticerci dead)/Number of cysticerci living at the start] × 100. Total survival probability for each time-interval was calculated by multiplying all survival probabilities at all time-intervals preceding that time.

ELISA TGFβ data were compared using a chi-squared test in three different conditions; all samples (before and after treatment) were included in the first one; in the second, only samples before treatment were included, and only samples after treatment were included in the last one. Contingency tables were generated with the following categories: response to cysticidal treatment (R and NR) and TGFβ concentration (low and high), considering values below or above the mean TGFβ concentration of 245 pg/dL. All data were recorded on InStat (GraphPad Software Inc., CA).

The inhibition effect of previous binding to TGFβ on receptor recognition by anti-TGFβ Type-I and -II receptor antibodies was analyzed by non-parametric ANOVA with Kruskal-Wallis post-test.

### Data Availability

The helminth genome datasets analyzed during the current study are publicly available in the GeneDB repository, www.genedb.org.

Any other datasets generated during and/or analyzed during the current study are available from the corresponding author on reasonable request.

## Results

### TGFβ Signaling Molecules are Present in *T. solium*

**Figure 1:**
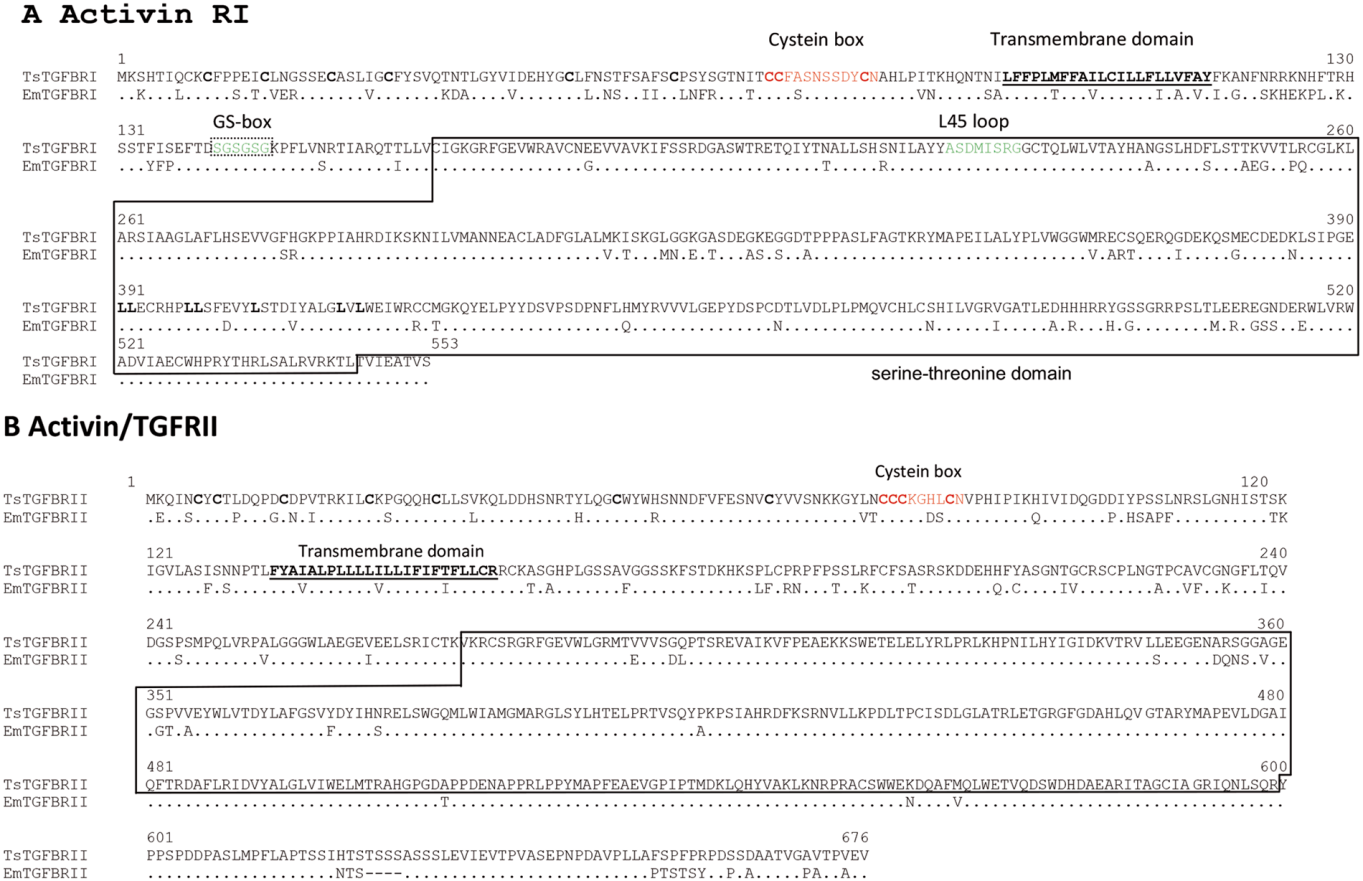
Protein sequence alignment for two TGFβ receptors of *Taenia solium* (Ts) and *Echinococcus multilocularis* (Em). (**A**) Activin receptor I for Ts and Em. (**B**) Activin receptor II for Em and Ts. Dashes represent amino acids that are not conserved in the sequence. Dots represent amino acids that are conserved in the sequence. Transmembrane domain is underlined and serine/threonine kinase domain is boxed. Cysteine box is depicted in red. For Type-I receptor, GS-box and L45 loop are indicated.

As shown in Fig. 1, both TGFβ receptor types, named TsTGFβRI and TsTGFβRII, were found in *T*. *solium* genome and transcriptome (www.taeniasolium.unam.mx and www.genedb.org/Homepage/Tsolium), as a 553- and a 676-amino acid long protein, respectively, showing characteristic features of these receptors like serine/threonine intracellular domains, transmembrane domains, and the cysteine box. Type-I receptor also contains characteristic elements of this group of molecules, like the GS-box (SGSGS) and the L45 loop (ASDMISRG). The typical signal peptide was absent in both TsTGFβRI and TsTGFβRII receptors. These receptors show a high amino acid sequence identity of 83% and 86% (Type-I and Type-II receptors, respectively) with the TGFβ family of receptors identified in closely related parasites of the genus *Echinococcus* ^20,21^. The 676-aa long sequence shows also a high identity (71%) with the activin Type-IIA receptor of *E*. *granulosus*.

Two additional molecules, 734- and 778 aa-long and with a high identify (93% and 90%) with the annotated TGFβ Type-I and with Tr3 (BMP Type-I receptor), respectively, of *E*. *granulosus* were also found. These sequences bear the characteristic motifs of the family of TGFβ Type-I receptors, but differ in several amino acids from the 553-aa protein described above (Supplementary Figure 1). No typical signal peptide was identified either.

In addition to the presence of TGFβ family receptors, several molecules of the TGFβ signaling pathway were found in the database of *T*. *solium* genome: the Smad anchor for receptor activation (SARA), signal transducers that in correspondence to those found in *Echinococcus multilocularis* were named TsSmadA and TsSmadC (equivalent to Smad2 and Smad3), TsSmadB (equivalent to Smad1,5,8), and the co-Smad named TsSmadD (equivalent to SMAD4), as well as the Transcription Factors E2F4/5, p300, p107, and DP1 (Supplementary Figure 2A–D), all of them show high homology with those found in *Echinococcus* sp.

A 503-aa-long ligand of the TGFβ family, which seems to correspond with *E*. *multilocularis* activin (ANCCV01195) (Supplementary Figure 3) according to its high identity (83%), was also found.

Table 1 shows the ID-numbers for genes and transcripts of the TGFβ signaling pathway found in the transcriptome database of *T*. *solium*.

**Table 1:** ID numbers for genes and transcripts of the TGFβ signaling pathway.

Protein name	ID of the proteins in the transcriptome *T. solium* database
Activin	TsM_000011500
TGFβ receptor Type-1	TsM_001248300
Activin receptor Type-1	TsM_000925600
Activin/TGFβ receptor Type-2A	TsM_000641800
TsTr3 (BMP) receptor Type-1	TsM_000081300
SARA	TsM_000334100
SmadA(2/3)	TsM_001006400
SmadB(1,5,8)	TsM_000781600
SmadC(2/3)	TsM_000602400
SmadD(4)	TsM_000635600
Transcription Factor E2F4/5	TsM_000874400
CREB binding protein	TsM_000337600
p107	TsM_001183400
DP1	TsM_001035400
Histone acetyltransferase p300	TsM_001200600

The sequences were found in the transcriptome database of *Taenia solium* (www.taeniasolium.unam.mx, and www.genedb.org/Homepage/Tsolium).

### Detection of TGFβ Type-I and Type-II Receptors in *T. solium* and *T. crassiceps* cysticerci

**Figure 2:**
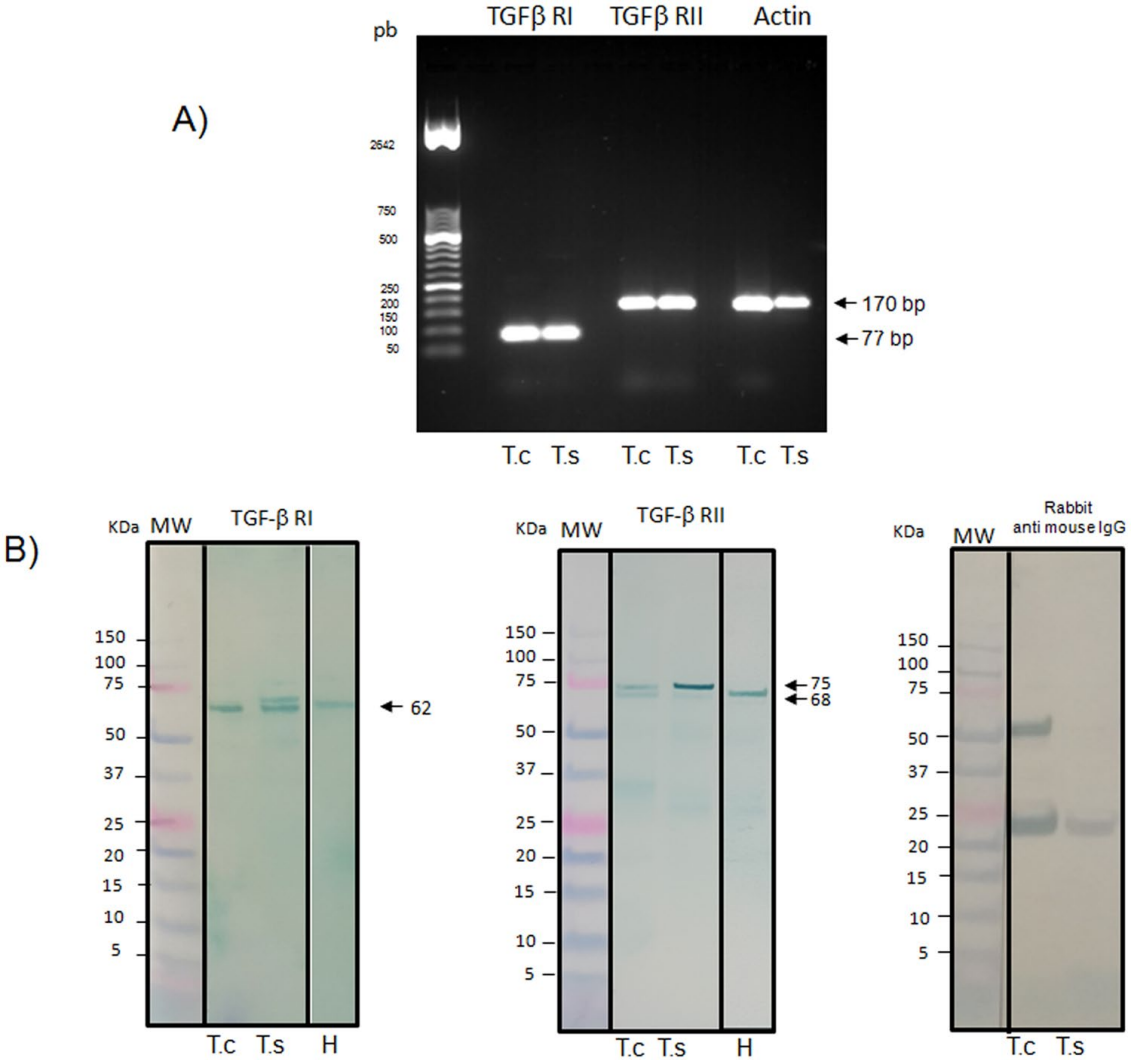
Identification of TGFβ Type-I and -II receptors by RT-PCR (**A**) and by Western bl.ot (**B**). (**A)** Expression of TGFβ Type-I and -II receptors in *Taenia solium* (Ts) and *T. crassiceps* (Tc) cysticerci. Amplified TGFβ Type-I and -II receptors obtained by RT-PCR from *T. solium* and *T. crassiceps* cysticerci are shown. A 50-pb ladder was use as DNA molecular Weight Marker; fragments amplified are 77 pb for TGFβ Type-I receptor, 170 pb for TGFβ Type-II receptor; actin beta-gamma (169 pb) was used as a positive control. (**B**) Western blot for the identification of putative TGFβ receptors in protein extracts from *T. crassiceps* cysticerci (T.c), *T. solium* (T.s), and human PMBCs (H), using polyclonal anti-human TGFβRI and TGFβRII antibodies. A rabbit anti-mouse IgG was included as a control. The white space in the human sample (H) for both TGFβ Type-I receptor TGFβ Type-II receptors mean that the blot was composed. The original blots are shown in Supplementary Figure 4.

Both TGFβ Type-I and-II receptors were found expressed in *T*. *solium* and *T*. *crassiceps* cysticerci by RT-PCR; the results are shown in Fig. 2A. The actin beta-gamma gene was used as positive control (TsM_000357600). In addition, to characterize the expression and localization of TGFβ receptors in *T*. *solium and T*. *crassiceps* cysticerci, polyclonal antibodies against human TGFβ Type-I and Type-II receptors were used in a western blot assay. As shown in Fig. 2B, antibodies against human TGFβ Type-IR recognized components of 62 kDa and 62- and 70-kDa for *T*. *crassiceps* and *T*. *solium*, respectively, that may correspond to Type-I receptors. On the other hand, antibodies against Type-II receptors recognized two major protein bands of 68- and 75 kDa, and one 75 kDa band for *T*. *crassiceps* and *T*. *solium*, respectively, which may correspond to the Type-II receptors. The rabbit anti-mouse IgG (control), recognized bands around 25- and 50 kDa, which most likely correspond to the light and heavy IgG antibodies (respectively) that are incorporated by the parasite^22^.

**Figure 3:**
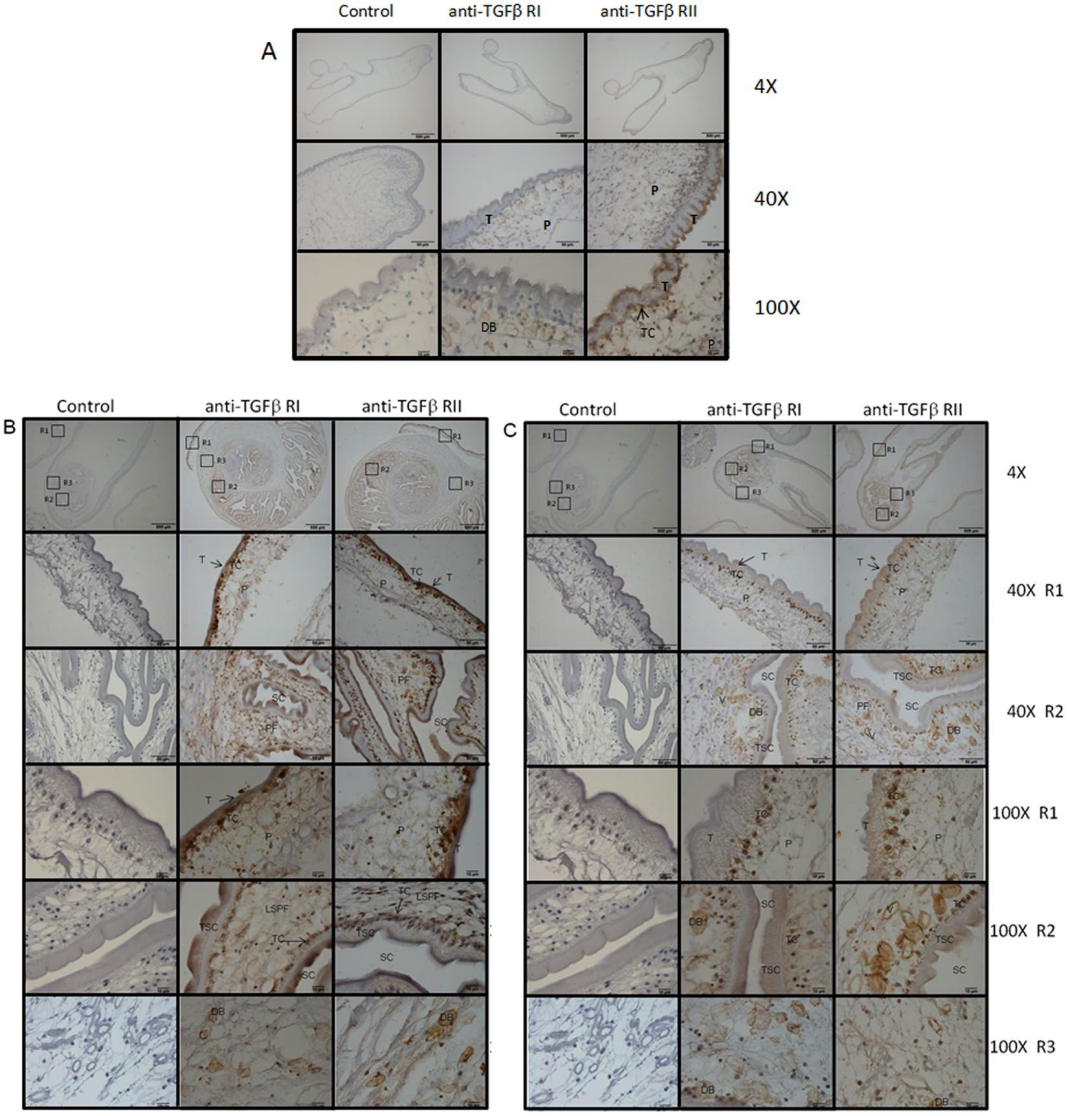
Immunolocalization of Type-I and -II TGFβ family receptors in *Taenia crassiceps* (**A**), brain-derived *T. solium* (**B**), and skeletal muscle-derived *T. solium* (**C**) cysticerci. Six-micrometer sections of both larval specimens reveal a strong binding to the tegument (T) and tegumental cells (Tc), and a less intense binding to parenchymal (P), dense bodies (DB), and vacuoles (V) both in *T. solium* and in *T. crassiceps* of rabbit anti-TGFβRI and RII antibodies. Subjacent nuclear layer (N), spiral canal (SC), parenchymal folds (PF), LSPF loose stroma of parenchymal folds, tegument spiral canal (TSC). Controls with non-immunized rabbit serum showed no reaction.

Native proteins of both receptors were searched on cysticerci by immunohistochemistry. Figure 3(A–C) shows the immunoreactions of both TGFβ Type-I and Type-II antibody receptors on *T*. *crassiceps* (A) and also on brain-derived (B) and skeletal muscle-derived *T*. *solium* (C) cysticerci. As shown, the signal was clearly more intense in *T*. *solium* than in *T*. *crassiceps*, and more so in cysticerci obtained from the brain of infected pigs. Immunoreactivity was found in the tegument (T), tegumental cells (Tc), parenchyma (P), dense bodies (DB), and vacuoles in both *T*. *solium* and *T*. *crassiceps* cysticerci.

### TGFβ Promotes *In Vitro* Cysticercal Growth and Survival

The TGFβ receptor was found expressed on the surface of *T*. *crassiceps* and *T*. *solium* cysticerci. Thus, the effect of TGFβ upon parasite growth was tested *in vitro*. As shown in Table 2, 0.01 and 0.1 ng/mL of TGFβ in culture medium caused a significant size increment of *T*. *crassiceps* cysticerci with respect to controls after day 13 of culture. Cysticerci cultured with 0.01 ng/mL of TGFβ started to show size increase at day 13 of culture. TGFβ-treated cysticerci started budding as soon as 6 days after culture, and 13 days later most cultured cysticerci (4 of 5 cysts) exhibited at least one bud each. Thus, a concentration of 0.01 ng/mL of TGFβ was quite efficient in inducing budding and parasite growth. All treated parasites remained alive during the follow-up period as assessed by temperature-induced motility, a previously employed method^19^.

**Table 2:** Effect of TGFβ on size and budding of *Taenia crassiceps* cysticerci.

Days of culture	Mean ± SD of the cysticerci area (pixels × 10^3^)				
	Concentration of TGFβ (ng/mL)				
	0 (RPMI)	0 (10% FBS)	0.001	0.01	0.1
0	13.1 ± 2 [0/0]^†^	15.1 ± 4.3 [0/0]	12.9 ± 3.1 [0/0]	14.4 ± 1.9 }[0/0]	13.1 ± 1.9 [0/0]
6	20.5 ± 2.8 (1.6) [0/0]	18.0 ± 7.4 (1.3) [2/2]	18.4 ± 5.6 (1.5) [1/1]	21.7 ± 2.9 (1.53) [2/2]	14.7 ± 3.2 (1.1) [0/0]
13	20.5 ± 3 (1.6) [1/1]	19.9 ± 3.1 (1.5) [3/6]	23.7 ± 6.8 (1.92) [3/4]	28.1 ± 4.4 (2.0)* [4/6]	24.1 ± 2.1 (1.9)* [2/4]
20	25.1 ± 5.8 (2.0) [1/1]	28.0 ± 5.6 (2.1) [3/7]	31.6 ± 8.8 (2.6) [3/6]	37.4 ± 3.8* (2.62) [4/6]	27.8 ± 3.6 (2.2) [2/4]

Mean ± SD of the area of five *Taenia crassiceps* cysticerci cultured by 20 days at different TGFβ concentrations. *Significant differences between each treatment and the control group at *P* < 0.05. ^†^Number of budding cysticerci/total number of buds in the five cysticerci. The numbers in parentheses show the fold increase in size with respect to day 0 of culture.

**Figure 4:**
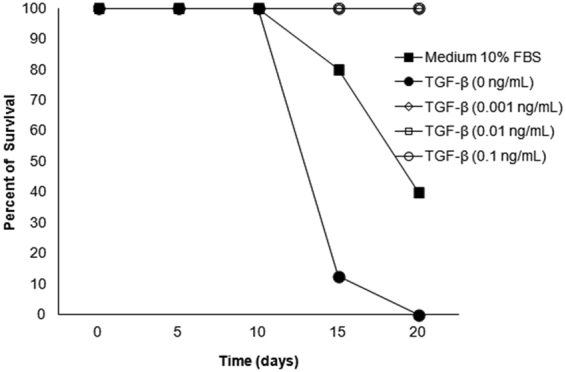
Effect of different TGFβ concentrations on the survival of *Taenia solium* cysticerci. Data are representative from three independent experiments.

Analogous experiments were performed with *T*. *solium* cysticerci. In this case, however, TGFβ treatment showed no effect upon cysticercus size, probably because *T*. *solium* cysticerci had transformed into tapeworms through an *in vitro* evagination process. Nevertheless, TGFβ treatment (in concentrations as low as 0.001 ng/mL–0.1 ng/mL) improved cysticercus survival rate, as shown in Fig. 4.

### TGFβ Binds to Parasite TsTGFβ RI and TsTGFβ RII Receptors

**Figure 5:**
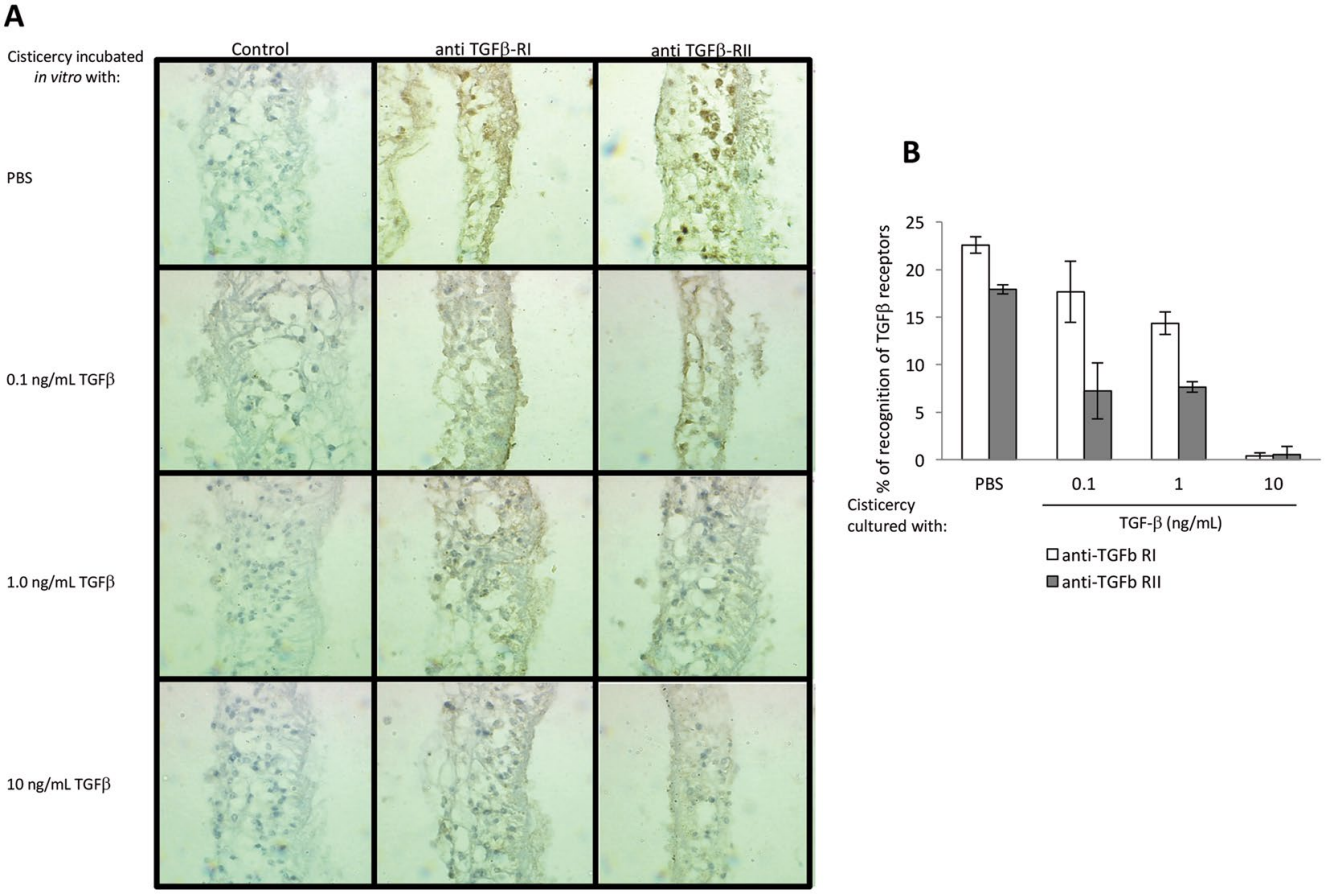
Inhibition of TGFβ receptor Type-I and -II-antibody recognition by TGFβ binding. *T. crassiceps* cysticerci were *in vitro* cultured with different concentrations of TGFβ for 30 minutes. Thereafter, cysticerci were extensively PBS-washed and fixed for immunohistochemistry studies for TGFβ receptor Type-I and -II-antibody recognition (**A**). (**B**) Mean ± SEM of the % of Type-I and -II TGFβ-antibody recognition. Image analysis for quantification of recognition was obtained as stated in Material and Methods. As increasing concentrations of TGFβ were employed, less recognition of both Type-I and -II receptors were observed (**B**). Significant differences using a non-parametric ANOVA with Kruskal-Wallis post-test.

The specificity of the binding of TGFβ to its receptor was evaluated in an inhibition experiment. *T*. *crassiceps* cysticerci were incubated either with or without TGFβ at different doses. After fixing for immunohistochemistry studies, the slides were incubated with anti-TGFβ Type-I and -II receptor antibodies; the intensity of bound antibodies for both receptors was measured using the MetaMorph software. As shown in Fig. 5, the addition of high amounts of TGFβ to cultured parasites reduced the expected recognition of both Type-I and -II TGFβ receptors.

### Increased TGFβ Levels in NC Patients Failing to Respond to Cysticidal Treatment

**Figure 6:**
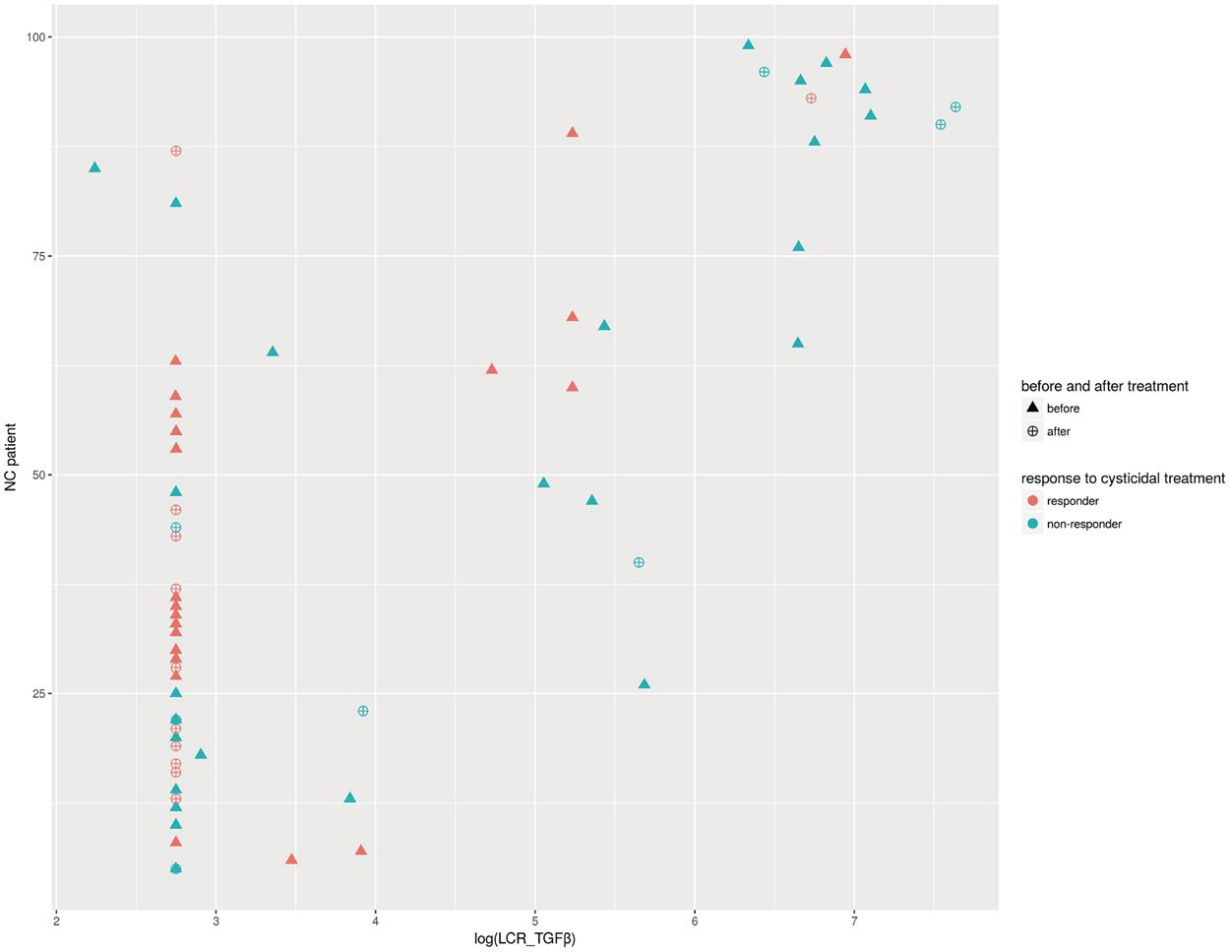
Correlation between cerebrospinal fluid TGFβ levels with the reduction in parasite size or number in responder and non-responder NC patients, before and after treatment. Individual logarithmic TGFβ levels before (triangle) and after (circle) treatment. Response to treatment was monitored by axial computed tomography (TAC). Patients were classified either as responders (orange circle) when the reduction in parasite size or number was higher than 50%, or non-responders (blue circle) when parasite load reduction was less than 50%.

TGFβ levels were measured before and after cysticidal treatment in the cerebrospinal fluid from non-responder (305.0 ± 405.0 and 499.0 ± 804.0, respectively) (mean ± SD) and responder patients (113.0 ± 248.0 and 90.3 ± 248.0, respectively). Before a new cycle of treatment was started, 28 patients were determined as non-responders, while 10 were found to respond to the treatment; after cysticidal treatment, 25 patients were found to respond to the treatment, while 13 failed to respond. Individual data of TGFβ levels of each patient are shown in Fig. 6. Before treatment, higher CSF-TGFβ levels were observed in non-responder NC patients (*P* = 0.03). Additionally, a correlation between CSF TGFβ levels and treatment response was observed when all samples (before and after treatment) were analyzed together (*P* = 0.003) and when only pre-treatment samples were considered (*P* = 0.03). No correlation was found after treatment (*P* = 0.1).

Other analysis considering gender and CSF-inflammation (cellularity) before and after treatment failed to show any difference.

## Discussion

A pro-inflammatory environment is present in the peritoneum of mice experimentally infected with *T*. *crassiceps* cysticerci^23,24^, as well as in the CSF from human patients harboring *T*. *solium* cysticerci, especially when parasites are lodged in the space at the base of the brain (SAB-NC)^3,25^. This infection-induced inflammatory response may surround vesicular, apparently healthy cysticerci. Thus, it is likely that some of the immune-inflammatory factors induced by the infection could promote parasite establishment, growth, and/or reproduction. Furthermore, various nematode and flatworm parasites exhibited different growth factor receptors^5^, highly conserved among helminths (i.e., TGFβ, EGF, and insulin receptor signaling pathways), apparently involved in parasite development^6,7,9^.

TGFβ is a cytokine that plays a role in pro-inflammatory as well as in anti-inflammatory responses^26^. The concomitant presence of TGFβ and IL6 favors a pro-inflammatory environment mediated by Th17^26^. In contrast, in an anti-inflammatory environment, TGFβ has been associated with inflammation control by inducing regulatory T cells (Tregs)^27,28^. In fact, parasite-secreted proteins promote Treg induction through the TGFβ pathway^29^. This fact highlights the importance of this cytokine during parasitic infections.

Thus, the relevance of TGFβ on the infection by *Taenia* sp. cysticerci and on the host-parasite relationship was assessed in this study. TGF-β/BMP superfamily signaling pathway-encoding genes were searched in the *T*. *solium* genome^30^ and compared with the genome of *E*. *multilocularis* (a closely related parasite for which TGFβ family members and signaling pathways have been widely characterized)^21^. Three Type-I TGFβ serine/threonine kinase family receptors were found. In contrast, only one Type-II TGFβ serine/threonine kinase family receptor was found. Motifs and domains that characterize Type-I and -II receptors, as well as in those proteins involved in the signaling pathway, are present in the sequences found in *T*. *solium* genome (Table 1, Fig. 1, and Supplementary Figures 1–3). As expected, a high homology of both *T*. *solium* Type-I and -II TGFβ receptors with Activin receptors of other helminths (i.e. *E*. *granulosus* and *Schistosoma mansoni*) was found. The repertoire of receptors found in *T*. *solium* has also been observed in other parasites, i.e., *S*. *mansoni*, *Caenorabditis elegans*, and *Brugia pahangi* ^31–33^. In the very well characterized TGFβ-signaling pathways, TGFβ or activin associates with Type-II receptors, which then recruits the corresponding Type-I receptor, which would lead to the activation and phosphorylation of the Smad2/3 that would form a protein complex with Smad4; finally, the translocation of this complex into the nucleus favors the expression of target genes. However, the presence of the MH1 domain in the Smads is required for the interaction of this protein complex with DNA. This MH1 domain was not found in Smad A/C but it was found in Smad B; the former is necessary for the TGFβ/Activin signaling, while the latter is needed for the signaling through BMP, similarly to what happens with *Echinoccocus* sp.^34^, but contrasting with the Smad2 of Platyhelminthes such as *S*. *mansoni*, which do have the MH1 domain^35^. However, our results show that TGFβ promotes the growth and survival of *Taenia* sp. cysticerci. Thus, it is feasible that the three different Type-I receptors in *Taenia* sp. may couple to the only Type-II receptor found, involving other protein complexes or mechanisms not yet identified in the signaling pathway to circumvent the absence of the MH1 domain in SmadA/C proteins; this hypothesis, nevertheless, still requires to be confirmed. The identification of phosphorylated proteins of the TGFβ family signaling pathway could help us to determine whether external TGFβ could mediate the observed changes via specific receptors. In an attempt to evaluate this, we used a polyclonal human anti-phospho-SMAD2/3; however, a high cross-reactivity was observed (data not shown). It is noteworthy that in parasites closely related to *T*. *solium*, i.e. *Caenorhabditis elegans*, *E*. *multilocularis* and *Schistosome mansoni*, a TGF-β family signaling pathway has been described^16,20,31,34,36^. Different processes were found to be regulated by the activation of this pathway in those parasites, such as body size, male tail development, embryo viability, and oocyte quality for *C*. *elegans*;^31,37–39^ and regulation of developmental processes such as body axis formation or regeneration and parasite development for *E*. *multilocularis* ^16^. For *Schistosoma spp*., which have been widely studied, fully functional components of the TGF-β signaling pathway have been found expressed for a certain time in various life cycle stages, indicating that TGF-β is involved in a number of developmental processes throughout the parasite life cycle^40^, such as embryonic development, production of vitellocytes in female blood-flukes^6^, and mitotic activity^40^, organ development, cellular growth and proliferation^41^, and eggshell formation^42^. These findings support the possibility that some components of the TGF-β family signaling pathway could also be regulating some of these processes in *Taenia spp*., a premise that requires further confirmation.

Genomic hints of Type-I and -II TsTGFβ receptors were confirmed by RT-PCR and by protein recognition using heterologous anti-TGFβRI and anti-TGFβRII antibodies. As shown in Fig. 2A, transcripts of both receptors were found in *T*. *solium* and *T*. *crassiceps* cysticerci. The sequences of the amplified RT-PCR products confirmed their expression. In accordance, human anti-TGFβRI and anti-TGFβRII antibodies were able to recognize proteins in *T*. *solium* cysticerci (Fig. 2B); namely, two 62- and 70-kDa bands for Type-I and one 75-kDa band for Type-II receptors were observed. Considering the close phylogenetic relation of *T*. *solium* with *T*. *crassiceps*, widely used as an experimental murine model of cysticercosis, we wondered if TGFβ receptors were also found in the latter cysticerci. Anti-human antibodies recognized one 62-kDa band for the Type-I and two 68- and 75-kDa bands for the Type-II TsTGFβ receptors in membrane extracts from *T*. *crassiceps* cysticerci. To ascertain the specificity of the polyclonal antibodies employed, extracts from human cells were assayed, and as shown in Fig. 2B, 62- and 68-kDa bands were observed for Type-I and -II receptors, respectively, in accordance with those found in *T*. *solium* and *T*. *crassiceps*. Thus, protein bands with molecular weights in the range of those observed in human cells were found in both cestodes. The presence of both receptors was also studied by immunohistochemistry. As shown in Fig. 3, both anti TGFβRI and TGFβRII receptor antibodies were attached in similar structures of both parasites, but more prominently in the tegument of *T. crassiceps* and also in *T*. *solium* cysticerci from brain and skeletal muscle of infected pigs (Fig. 3B,C). This anatomic localization in cysticercus periphery is compatible with the accessibility of the host’s molecules and cells: a fact that could be involved in cysticercal growth and differentiation, as it occurs in other parasites^43^. The presence of putative TGFβ receptors and several molecules involved in the signaling pathways poses new questions on their relevance in host-parasite interactions. A more intense immuno-detection of both family receptors was found in *T*. *solium* cysticerci than in *T*. *crassiceps*. One finding that merits comments is the higher immuno-detection of both family receptors in *T*. *solium* cysticerci recovered from the brain than in those from skeletal muscle. This higher expression could be due to the higher TGFβ levels in pig CSF than in serum, as observed in neurocysticercosis patients^44^, possible due by the expression of the corresponding ligand. Thus, cerebral cysticerci would be immersed in a compartment enriched with this growth factor, and this environment could be related with the higher resistance to damage in cerebral cysticerci with respect to those found in muscles. The receptors were also immunolocated in cysticercal dense bodies and vacuoles; however, their relevance in the physiology of the parasite needs to be studied.

TGFβ regulates cellular processes such as proliferation, differentiation, motility, adhesion, organization, tissue restoration, embryonic development, and programmed cell death in many physiological systems, and its signaling pathway is highly conserved from invertebrates to humans^45^. The presence of a TGFβ signaling pathway in *T*. *solium* suggests that several of the above-mentioned processes could be present in this parasite, and moreover, that host- or parasite-TGFβ family proteins could modulate these responses. Indeed, a homologue of the TGFβ family protein was found by *in silico* analysis of the *T*. *solium* secretome^46^, and this suggests that some of these TGFβ-mediated processes could be modulated by parasite proteins. However, there is also a possibility that host proteins of the TGFβ family could be employed by the parasite. In fact, in this study, recombinant human TGFβ was found to be able to promote *in vitro* the growth and reproduction of *T*. *crassiceps* cysticerci (Table 2). TGFβ failed to induce significant changes in the size of *T*. *solium* cysticerci, but it had a clear effect upon parasite survival (Fig. 4). The effect observed on both cysticercus species could be due to the internalization of TGFβ via endocytosis as a regulatory event^47^. However, two facts reinforce the possibility that effects are mediated by its interaction with parasite receptors. The first is the lower antibody recognition of both Type-I and -II parasite receptors when cysticerci were cultured with increasing levels of TGFβ (Fig. 5), suggesting that TGFβ could bind the Type-II receptor, avoiding antibody recognition; the complex TGFβ-TsTGFβRII receptor would recruit the Type-I receptor, forming a complex which would also prevent the Type-I receptor antibody to be bound. The second fact is that a significantly lower effect on parasite growth and survival was found when cysticerci were cultured with fetal bovine serum (FBS) with respect to cysts cultured with TGFβ, even though the serum contained other proteins that could also be internalized by endocytosis. With respect to the first proposed mechanism, a non-dose-dependent effect was observed in *T*. *crassiceps*, which could be attributed to a saturation in TGFβ receptor binding, as previously reported^47^. Interestingly, the effect of TGFβ upon *Taenia* sp. cysticerci differs from *E*. *multilocularis*, in which no physiological response has been observed^21^, pointing to a relevant difference between these two closely related cestodes; these dissimilarities would merit further studies.

In previous works, our group reported increased inflammatory features in NC, particularly higher IgG, IL1β, IL5, and IL6 levels correlated with severity^3,48^, while higher TGFβ levels were found in most severe patients^44^. These responses could be the result of an effort by immunocompetent NC patients to control the increased inflammatory response that gives rise to the production of a parasite-related immune-modulating factor.

Based on these *in vitro* evidences, the relevance of TGFβ for cysticercus permanence and growth *in vivo* has been considered. In severe NC cases caused by the establishment of cysticerci in the subarachnoid space of the base of the brain, cysticerci are imbedded in TGFβ-enriched cerebrospinal fluid. It is then plausible that TGFβ could promote a more permissive environment for parasite survival, which in turn may result in the ineffectiveness of cysticidal drugs. The finding that non-responding SAB-NC patients exhibited significantly higher TGFβ levels in CSF than responder patients before any treatment supports this possibility. Thus, other differences among these patients could affect the responsiveness to the cysticidal treatment. Nevertheless, considering the possible relevance of this finding in NC severe cases, it should be further explored since it could lead to new approaches to increase the effectiveness of cysticidal treatments, such as the use of specific monoclonal antibodies against TGFβ during treatment.

Altogether, the findings herein reported point to TGFβ as a cysticercal growth and survival factor, which could play a role in the lack of effectiveness of cysticidal treatment.

## Electronic supplementary material

Supplementary Figures

## References

[1] Sciutto E (2000). *Taenia solium* disease in humans and pigs: an ancient parasitosis disease rooted in developing countries and emerging as a major health problem of global dimensions. Microbes Infect.

[2] Terrazas LI, Bojalil R, Govezensky T, Larralde C (1998). Shift from an early protective Th1-type immune response to a late permissive Th2-type response in murine cysticercosis (*Taenia crassiceps*). J Parasitol.

[3] Sáenz B (2012). Neurocysticercosis: local and systemic immune-inflammatory features related to severity. Med Microbiol Immunol.

[4] Brehm K, Spiliotis M (2008). The influence of host hormones and cytokines on *Echinococcus multilocularis* signalling and development. Parasite.

[5] Dissous C, Khayath N, Vicogne J, Capron M (2006). Growth factor receptors in helminth parasites: signalling and host-parasite relationships. FEBS Lett.

[6] Freitas TC, Jung E, Pearce EJ (2007). TGF-beta signaling controls embryo development in the parasitic flatworm *Schistosoma mansoni*. PLoS Pathog.

[7] Spiliotis M, Kroner A, Brehm K (2003). Identification, molecular characterization and expression of the gene encoding the epidermal growth factor receptor orthologue from the fox-tapeworm *Echinococcus multilocularis*. Gene.

[8] Morales-Montor J, Larralde C (2005). The role of sex steroids in the complex physiology of the host-parasite relationship: the case of the larval cestode of *Taenia crassiceps*. Parasitology.

[9] Konrad C, Kroner A, Spiliotis M, Zavala-Góngora R, Brehm K (2003). Identification and molecular characterisation of a gene encoding a member of the insulin receptor family in *Echinococcus multilocularis*. Int J Parasitol.

[10] Katz LH (2013). Targeting TGF-β signaling in cancer. Expert Opin Ther Targets.

[11] Shimmi O, Newfeld SJ (2013). New insights into extracellular and post-translational regulation of TGF-β family signalling pathways. J Biochem.

[12] Restrepo BI (2001). Brain granulomas in neurocysticercosis patients are associated with a Th1 and Th2 profile. Infect Immun.

[13] Hirata M, Hirata K, Hara T, Kawabuchi M, Fukuma T (2005). Expression of TGF-beta-like molecules in the life cycle of *Schistosoma japonicum*. Parasitol Res.

[14] Freitas TC, Jung E, Pearce EJ (2009). A bone morphogenetic protein homologue in the parasitic flatworm. Schistosoma mansoni. Int J Parasitol.

[15] Liu R (2013). Cloning and characterization of a bone morphogenetic protein homologue of *Schistosoma japonicum*. Exp Parasitol.

[16] Epping K, Brehm K (2011). *Echinococcus multilocularis*: molecular characterization of EmSmadE, a novel BR-Smad involved in TGF-β and BMP signaling. Exp Parasitol.

[17] Larralde C (1989). Deciphering western blots of tapeworm antigens (*Taenia solium*, *Echinococcus granulosus*, and *Taenia crassiceps*) reacting with sera from neurocysticercosis and hydatid disease patients. Am J Trop Med Hyg.

[18] Rosas G (2002). Protective immunity against *Taenia crassiceps* murine cysticercosis induced by DNA vaccination with a *Taenia saginata* tegument antigen. Microbes Infect.

[19] Martínez-González JJ, Guevara-Flores A, Alvarez G, Rendón-Gómez JL, Del Arenal IP (2010). *In vitro* killing action of auranofin on *Taenia crassiceps* metacestode (cysticerci) and inactivation of thioredoxin-glutathione reductase (TGR). Parasitol Res.

[20] Zavala-Góngora R, Kroner A, Bernthaler P, Knaus P, Brehm K (2006). A member of the transforming growth factor-beta receptor family from *Echinococcus multilocularis* is activated by human bone morphogenetic protein 2. Mol Biochem Parasitol.

[21] Brehm K, Koziol U (2017). Echinococcus-Host Interactions at Cellular and Molecular Levels. Adv Parasitol.

[22] Navarrete-Perea J (2014). Identification and quantification of host proteins in the vesicular fluid of porcine *Taenia solium* cysticerci. Exp Parasitol.

[23] Peón AN, Espinoza-Jiménez A, Terrazas LI (2013). Immunoregulation by *Taenia crassiceps* and its antigens. Biomed Res Int.

[24] Terrazas LI (2008). The complex role of pro- and anti-inflammatory cytokines in cysticercosis: immunological lessons from experimental and natural hosts. Curr Top Med Chem.

[25] Matos-Silva H (2012). Experimental encephalitis caused by *Taenia crassiceps* cysticerci in mice. Arq Neuropsiquiatr.

[26] Bettelli E (2006). Reciprocal developmental pathways for the generation of pathogenic effector TH17 and regulatory T cells. Nature.

[27] Yamagiwa S, Gray JD, Hashimoto S, Horwitz DA (2001). A role for TGF-beta in the generation and expansion of CD4+ CD25+ regulatory T cells from human peripheral blood. J Immunol.

[28] Pyzik M, Piccirillo CA (2007). TGF-beta1 modulates Foxp3 expression and regulatory activity in distinct CD4+ T cell subsets. J Leukoc Biol.

[29] Grainger JR (2010). Helminth secretions induce de novo T cell Foxp3 expression and regulatory function through the TGF-β pathway. J Exp Med.

[30] Tsai IJ (2013). The genomes of four tapeworm species reveal adaptations to parasitism. Nature.

[31] Savage-Dunn C (2001). Targets of TGF beta-related signaling in *Caenorhabditis elegans*. Cytokine Growth Factor Rev.

[32] Gomez-Escobar N, van den Biggelaar A, Maizels R (1997). A member of the TGF-beta receptor gene family in the parasitic nematode *Brugia pahangi*. Gene.

[33] Forrester SG, Warfel PW, Pearce EJ (2004). Tegumental expression of a novel type II receptor serine/threonine kinase (SmRK2) in *Schistosoma mansoni*. Mol Biochem Parasitol.

[34] Zavala-Góngora R, Derrer B, Gelmedin V, Knaus P, Brehm K (2008). Molecular characterisation of a second structurally unusual AR-Smad without an MH1 domain and a Smad4 orthologue from *Echinococcus multilocularis*. Int J Parasitol.

[35] Osman A, Niles EG, LoVerde PT (2001). Identification and characterization of a Smad2 homologue from *Schistosoma mansoni*, a transforming growth factor-beta signal transducer. J Biol Chem.

[36] Gelmedin V, Zavala-Góngora R, Fernández C, Brehm K (2005). *Echinococcus multilocularis*: cloning and characterization of a member of the SNW/SKIP family of transcriptional coregulators. Exp Parasitol.

[37] Luo S, Kleemann GA, Ashraf JM, Shaw WM, Murphy CT (2010). TGF-β and insulin signaling regulate reproductive aging via oocyte and germline quality maintenance. Cell.

[38] McGehee AM, Moss BJ, Juo P (2015). The DAF-7/TGF-β signaling pathway regulates abundance of the *Caenorhabditis elegans* glutamate receptor GLR-1. Mol Cell Neurosci.

[39] Dineen A, Gaudet J (2014). TGF-β signaling can act from multiple tissues to regulate *C. elegans* body size. BMC Dev Biol.

[40] Loverde PT, Osman A, Hinck A (2007). *Schistosoma mansoni*: TGF-beta signaling pathways. Exp Parasitol.

[41] Oliveira KC, Carvalho ML, Verjovski-Almeida S, LoVerde PT (2012). Effect of human TGF-β on the gene expression profile of *Schistosoma mansoni* adult worms. Mol Biochem Parasitol.

[42] Buro C (2013). Transcriptome analyses of inhibitor-treated schistosome females provide evidence for cooperating Src-kinase and TGFβ receptor pathways controlling mitosis and eggshell formation. PLoS Pathog.

[43] Davies SJ, Shoemaker CB, Pearce EJ (1998). A divergent member of the transforming growth factor beta receptor family from *Schistosoma mansoni* is expressed on the parasite surface membrane. J Biol Chem.

[44] Adalid-Peralta L (2012). Human neurocysticercosis: *in vivo* expansion of peripheral regulatory T cells and their recruitment in the central nervous system. J Parasitol.

[45] Gumienny, T. L. & Savage-Dunn, C. TGF-β signaling in *C*. *elegans*. *WormBook*, 1–34, doi:10.1895/wormbook.1.22.2 (2013). 10.1895/wormbook.1.22.2 PMC5081272 23908056

[46] Gomez S (2015). Genome analysis of Excretory/Secretory proteins in *Taenia solium* reveals their Abundance of Antigenic Regions (AAR). Sci Rep.

[47] Kells AF, Schwartz HS, Bascom CC, Hoover RL (1992). Identification and analysis of transforming growth factor beta receptors on primary osteoblast-enriched cultures derived from adult human bone. Connect Tissue Res.

[48] Chavarría A (2005). Relationship between the clinical heterogeneity of neurocysticercosis and the immune-inflammatory profiles. Clin Immunol.

