# Supplementary material for: Effect of Transforming Growth Factor-β upon Taenia solium and Taenia crassiceps Cysticerci
Source: Sci Rep. 2017 Sep 27;7:12345. doi: 10.1038/s41598-017-12202-z (PMC5617888; doi:10.1038/s41598-017-12202-z)

Effect of Transforming Growth Factor-β upon *Taenia crassiceps and Taenia solium* cysticerci

Laura Adalid-Peralta, Gabriela Rosas, Asiel Arce-Sillas, Raúl J Bobes, Graciela Cárdenas, Marisela Hernández, Celeste Trejo, Gabriela Meneses, Beatriz Hernández, Karel Estrada, Agnes Fleury, Juan P Laclette, Carlos Larralde, Edda Sciutto, Gladis Fragoso

Supplementary Figure 1

A TGFβ RI

```
                                                                                                   Cystein box          Transmembrane domain
           1                                                                                                                            130
TsTGFbRI   MSVDELGTWRRYIDALLQNFNDEESNNAYYRQIWEEPFCCTISPGERCIIKLINEPNVPPHRLYEMGCLRQKDFWKPFSCESANMRCCNSSFCNLPLKEELSKLIVEVPKDNTLIVILSIFLVLAVLMLV
EmTGFbRI   ..................................................................................................................................

           131                                                                                                                          260
TsTGFbRI   AGWCFWRCRDKADWRKSSQAPVSMIGAGCIGDATGHTSPSDTHPWHMAECGASGASMVSAIPTATTTANASAVAVNGGVLNATAANSGVSVATTLIDSVPLHPSRTPFLMASSSISSVIAVRPPRGLGAI
EmTGFbRI   ..................................................................................................................................

                                                                     GS-box
           261                                                                                                                          390
TsTGFbRI   PTGVSSYHNSIGGSTVVRASEVVGLGGGGASYTAASGGASMLPTSCSAPPGAGGTLEITLSGSGSGAGQPLLVERTVARQVTLSARIGEGRYGEVWLGRLHGDQVAVKIFSSRNENSWIREKEIYETATL
EmTGFbRI   ..................................................................................................................................

                      L45 loop
           391                                                                                                                          520
TsTGFbRI   RHSNILGFIAADNKDNGISTELWLIAEYHRLGSLYEFLQSHAFTLPALIKMASSIANGLAHLHMAIVGTSGKPPIAHRDLKSRNILVKADGECCIGDLGFAVKYDSLTGRVDIGHNTERIGTKRYMAPEV
EmTGFbRI   ..................................................................................................................................

           521                                                                                                                          650
TsTGFbRI   LDNTLYAHSFDAYMQADIYSLGLVFWEMTRRVYYANLYGPEEYQLPYQDNVGPDPLIEEMKSVVCELGIRPHLPLVWQTDASLRLLYHIMTECWFANPSHRLSAMRIKKDLANQRQQLGSPRQPGETPLP
EmTGFbRI   ..................................................................................................................................

           651                                                                                  734
TsTGFbRI   PHILRQQPSSLDALPPGVFVYRSVEAASAASNANTSTLETNQVGEAEAEAEEALLASGGPTIQPPNVVATAAVSGGRGNLGYCX
EmTGFbRI   ....................................................................................
```

B TsTr3 (BMPRI)

```
                                                                                                                           Cystein box
        1                                                                                                                            130
TsTr3   MFAFCLFLLLSSAKLSTANQEECLKLLCTADESDCHPCTGMSADELGTWRRYIDALLQSFNDEEN-NAYYRKIREEPFCCRISPGERCIIKLIKEPNVPPYRLYEMGCLRQKDFWKPFSCKSANTRCCNS
EmTr3   ...LS.-..FS...PS...RK.......NP........A...V...............N.....SS.....Q.W......T.................................E...M.....

        131                                                                                                                          260
TsTr3   SFCNLPLKEELAKLIDEVPKDNTLIVILSIFLVLAVLMLIAGWCFWRCRDKA----AAQMPVSMVGAGCIGDAAGHTSPSDPRPWHMVECGGSGASMVSAIPTATTAASASVVAVNGGVLNVTAANSGAS
EmTr3   ..........S...V.....................V. .........DWRKSS.A....I.......T.......TH...A...A..............T.NA.A.........A......V.

                                                                                                                   GS-box
        261                                                                                                                          390
TsTr3   VATTLIDSIPLHPSRAPFLMASSSISSVVAVRSPRGLGAVPTGVSSYHNSIGGSTVVRGPEIVGHGSGCGGGGASYTAASGGASMLPASCSAPPGAGGTLEITLSGSGSGAGQPLLVERTVARQVTLSAR
EmTr3   .......V......T............I...P......I..................AS.V.G----L..................T.......................................

                                                                  L45 loop
        391                                                                                                                          520
TsTr3   IGEGRYGEVWLGRLHGDQVAVKIFSSRNENSWIREKEIYETATLRHSNILGFIAADNKDNGISTELWLIAEYHRLGSLYEFLQTHAFTLPALIKMASSIANGLAHLHMAIVGTSGKPPIAHRDLKSRNIL
EmTr3   ..................................................................................................................................

        521                                                                                                                          650
TsTr3   VKADGECCIGDLGFAVKYDSLTGRVDTGHNTERVGTKRYMAPEVLDNTLCAHSFDAYMQADIYSLGLVFWEMTRRVYYANLYGPEEYQLPYQDNVGPDPLIEEMKSVVCELGIRPHLPLVWQSDASLRLL
EmTr3   ..........................I......I...............Y.......................................................................T.......

        651                                                                                                                      778
TsTr3   YHIMTECWSANPSHRLSAMRIKKDLANQRQQLGSPRQPGETPLPPHILRQQPSSLDALPPGVFVYRSAEAASATNNANTSTLVTNQV--EEAEVEEALLASGGPTVQPPNVVATAAMSGGRGNLGYCX
EmTr3   ........F.................. ....................................V.....AS.......E....GEA...AE..........I..........V..........
```

Supplementary Figure 2

SmadA (Smad2)

A

```
           1                                                                                                                          130
TsSmadA    MSNIALHRAASDYTSSSDSSRLVSPPSVASGASGSTFSDSLTVLPPHSGTLYGDPLVQPYDRQQVMPVGDSR--RWSHLEGECERQQHFDEYSTSNLNVENTQSSNPNPPVPSHPANVPLTPSLKNWCSV
EmSmadA    .......CTT.....................---.......AL...N..A.....................NNG..............C.D...N....G....P.S.....P..T..............

           131                                                                                                                        260
TsSmadA    FYYELNNRVGDVFHASKPKFTVDGFTAPSLGTERFSLGGLSHVNRPPQVDMTRRHIGRGLNLLYISGEVFVECLSDAAIFVQSPSCNRLNNWHPATVVKVPPRCNLRVFDNREFADLLSQSVTRNYETVF
EmSmadA    ...................................................................................................................E..............

                            L3loop
           261                                                 322
TsSmadA    SLTHMCFIRISFVKGWGADYRRQTITSTPCWIELHLNEPLKWLDRVLQEMGSPSTPCTSVSX
EmSmadA    .............................................................
```

SmadB (Smad1,5,8)

B

```
           1                                                                                                                          130

TsSmadB    MSTPISSLNSLFSFTSPAVKRLLGWKQGDEEEKWAEKAVDSLVKKLKKKKGALESLEKALSNPGEPSECVTIPRSLDGRLQVSHRKGLPHVIYCRVWRWPDLQSHHELKALDICRFPFSAKENEVCINPY
EmSmadB    ...S.........................................................................................................P....................

           131                                                                                                                        260
TsSmadB    HYKRVESPVLPPVLVPRHSEYPCMGSVAAGPSTDSTRISVMPLQATPLLMASSYGQLTSSEPSMPYNVTYPQGFTQTSPLPPATPVSSGASPSTSLPPPSDTHPVNYQEPKYWCSIVYYELNTRVGEAFF
EmSmadB    ...........................S...PA......I.....A...V......P..GG........................I..................I.........................

           261                                                                                                                        390
TsSmadB    ASQPSIVIDGFTDPSNNSDRFCLGLLSNVNRNSTIENTRRHIGKGVHLYYVGGEVFAECLSDSSIFVQSRNCNHRHNFNLTTVCKIPPGCSLKIFSNQEFAQLLRRTVSHGFEAVYELTKMCTIRMSFVK
EmSmadB    ..................................................................................................................................

           391     L3loop                            438
TsSmadB    GWGAEYHRQDVTSTPCWVEIHLNGPLQWLDRVLTQMGTPRNPISSVSX
EmSmadB    ...............................................
```

SmadC (Smad3)

C

```
           1                                                                                                                          130
TsSmadC                                       MAMKSSPPTTSSPPQMSPTNCSKTTGAGTGKLSEGGESSAGPALTTDALSCTAPGSSSSGGGSGDATSTGGLDSRSPGALSRDENCLSEDNDTMD
EmSmadC    MGPSYRSSGDDSTTQSSSPHTGSMLSYLSHGLKNS..I.P...S....TLT.SA.............D..........G...................................................

           131                                                                                                                        260
TsSmadC    TGLDTLNDITIDDSAHTSVAYQEPDYWCSVYYYETNTRVGDTFYCASPCLTVDGFTDPSRENRFCLGLLSNVNRGHQIELTRRHIGHGVALYYIGGEVFAECLSDSAIFVQSPNCNHMYNWHPATVCKIP
EmSmadC    ...........G.............C........................................................................................................

                                                      L3loop
           261                                                                                      351
TsSmadC    PGCNLKIFNNQHFAALLKESVNNGFEAVYALTNMCTIRISFVKGWGAEYRRQTVTSTPCWIELHLNGPLQWLDRVLSQMGSPNHPCTSVSX
EmSmadC    ..........................................................................................
```

Supplementary Figure 2

SmadD (Smad4)

D

```
         1                                                                                                                              130
TsSmadD  MVLDATTACLSSHSMVMKILGTDHEPSHSQSRSLTQSRGHLSVNTSTSSDACMNIVHSLMCHRKGGESEEFSKFAIESLIKKLKDRRDELDALIVAVTSNGATQTSCVTIQRTLDSRMQIAGRKCFPHLI
EgSmadD  .................................I.......A....................................................A...................................

         131                                                                                                                            260
TsSmadD  YARLWRWSDAHKTELRHLPFCHFGFDKKLDWVCVNPYHYERTVSSALDISSLALSPPVERRKGKVACTRVHNLSSGVEGEEVFNKQRRHSSELQHSDTSHPTPTLRLSVQSLLDESPLCVPALSGGVKQE
EgSmadD  ...............................................................-------------..E...L............P.....AS..................T....I.P.

         261                                                                                                                            390
TsSmadD  PTKNSTIAYEPSHRQQQQPTSTAATVTTTTANPLSLENVDAATANLLNLMQWVSNANATFAAAVAAGGGGVGAHSAPPPPPPASLQALPSSHPTSSTDEASAVALDAAETAALLDPIAASGGAPAPVLYP
EgSmadD  .T.........A.--.......V.SA...P-.....................................S...T.......R.PPV....AL. .....................................

         391                                                                                                                            520
TsSmadD  SQCSGGSGGTNNGQPAFTAGSSSSSYGRASQQQQQPVSSSSTASLGGGWGGGG-APGGQPPPMLPPNPPPSGSLQPSQLTTQRPPEYWCNIAYFELDQQVGELFKVPSHYTRVIVDGYTDPSSRNRFCLG
EgSmadD  ..............S...................-....... P.........G............................................. ..............................

         521                                                                                                                            650
TsSmadD  QLSNVHRSEQSEKSRLYIGKGVELDIVGEGDVWIRCLSEFSIFVQSYYLDREAGRAPGDAVHKIYPGAYIKVFDIRQCHEQMRHLAHMTQAAAMRQAAAVAG----------------------------
EgSmadD  ..................................... ................................................................SMQMGAVADAAAAASAAAAAATAPMGTC

         651                                                                                  739
TsSmadD  ------VDDFRRLCNLRLSFVKGWGPDYPRHDIKETPCWWIEIQLHRPLQLLDEVLQAMPLNDLKPTRHFFYYQNQLQQPPPSQHLAKRX
EgSmadD  EAAGVG..........................................................................P--......
```

L3loop

Supplementary Figure 3

Activin

```
                 1                                                                                                  100
TsTGFβfamily     MTSTVPMKCGIALIALALIMLCNCPPSHALFRQPAIMEGVRLGDQDQEEEE.IWVGPVPKIEDNEEHTVDDVETKKTVDEEEEREREAKRRA...EEEEF
EmTGFβfamily     ..I.T......V.V.......GS..LI..........D.TL.EVP......RM..D.T......DGND....G...KLE.T...........DEE.....

                 101                                                                                                200
TsTGFβfamily     TRQIHIEKFKRSLLQRLHLTAPPDFSRHGGMANRTHGSRMLRSLPLALQGRLLNQMRAEDGAAEPPPDRTDERETLILLKHLHWKLPKVASATFGIEMAD
EmTGFβfamily     E.L........T..K.....S..................V............................................................

                 201                                                                                                300
TsTGFβfamily     DVDPSRIKSAFLRFETKNPMLKGEQLEVWEIFMTSQEEEEELSKKAVDKQSLEHYNNLTWTLDRQSAGYTSLPAPAVIRGSLGSPAEHRRTGSIRIRPGR
EmTGFβfamily     .I.....Q...............QHV....V...PS...GKMTNA...QP...Q......MFE..PTK...........R.....V..............

                 301                                                                                                400
TsTGFβfamily     LAETFVPSCPGLVQVTFEISGSFAQWMSHRRRMPLMRKLVRSILVVCPKCSSHVDPVDVNKGILEIHHRNVVRRIRRSLDSNGSHHVPIGNPCSPKGHKF
EmTGFβfamily     ..........................................LI....D.........................T.....T.S.Q...............

                 401                                                                                                500
TsTGFβfamily     SCCTQPFSLNLEDVGWNNNWILHPKTVEPNYCHGSCQADGIQKTPHSDLMHMYRSQNYDHLSEVQREAMLSCCHPVKMASTSVLYVDPDNELHMDTLHNII
EmTGFβfamily     ..................................................L.................................................

                 501
TsTGFβfamily     VLECGCS
EmTGFβfamily     .......
```

Supplementary Figure 4A

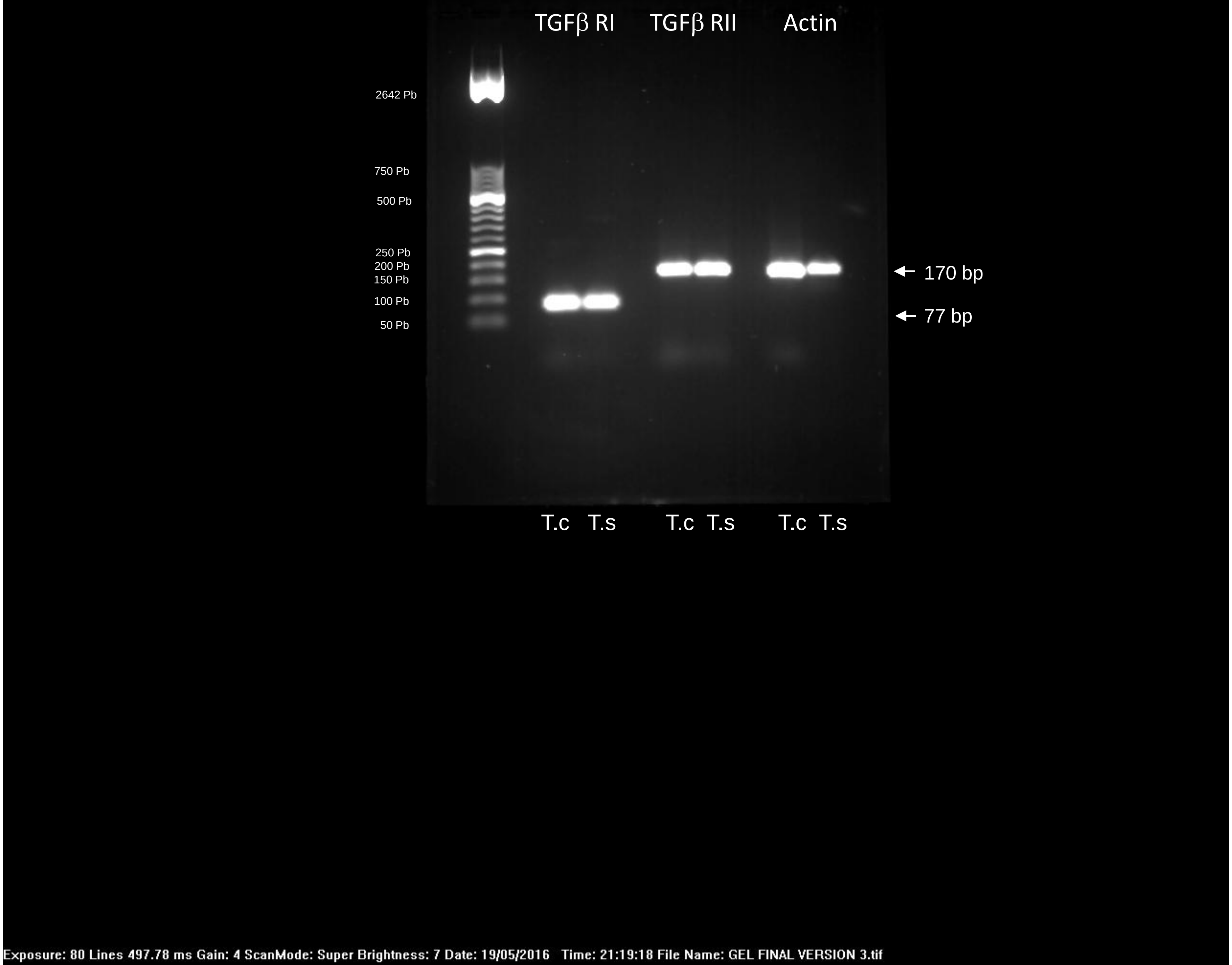

Supplementary Figure 4B

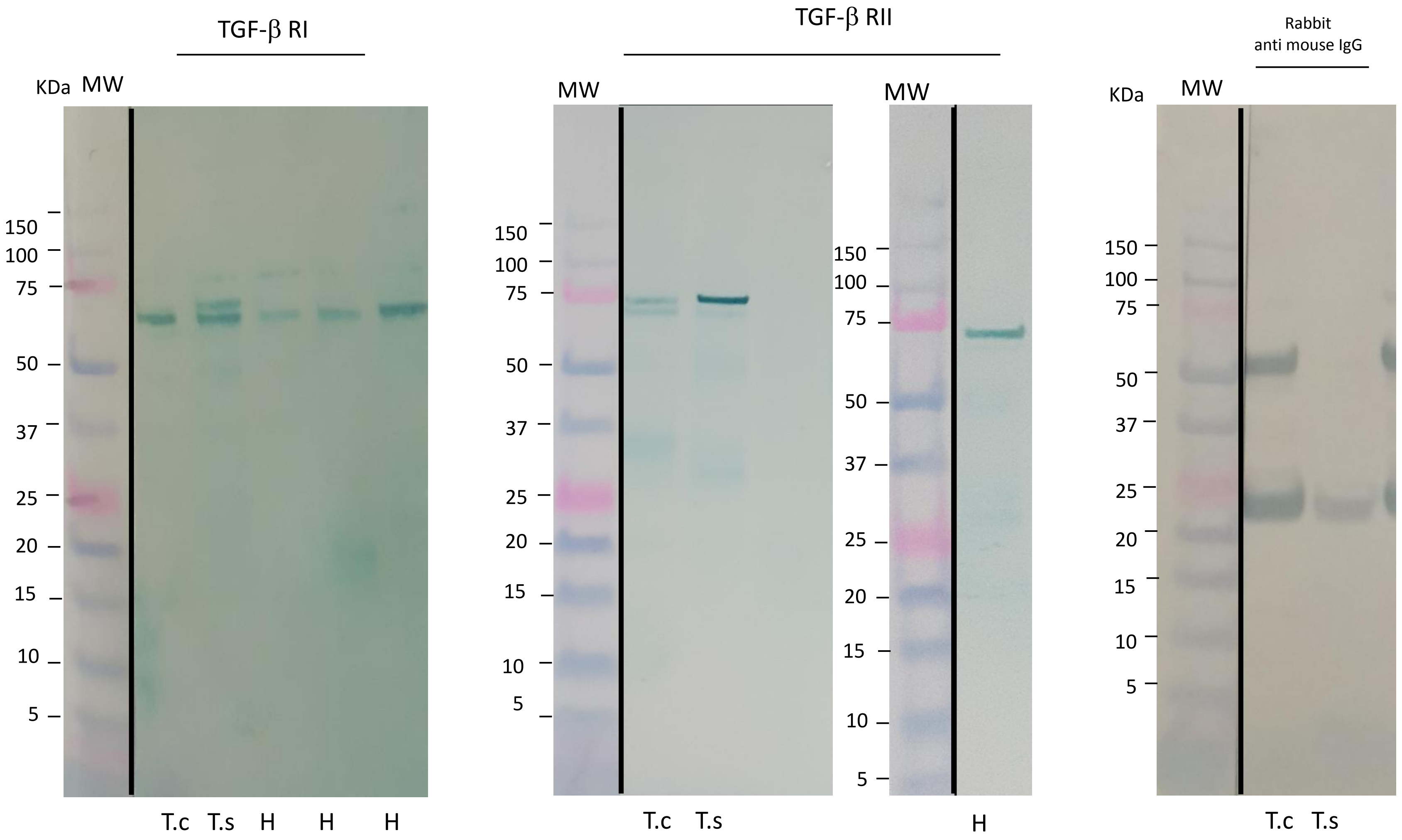

Supplement: Supplementary file 1 — Supplementary Figures [file 41598_2017_12202_MOESM1_ESM.pdf]
